# The Mental Health of Caregivers and Their Patients With Dementia During the COVID-19 Pandemic: A Systematic Review

**DOI:** 10.3389/fpsyg.2021.782833

**Published:** 2021-12-24

**Authors:** Elvira Anna Carbone, Renato de Filippis, Roberta Roberti, Marianna Rania, Laura Destefano, Emilio Russo, Giovambattista De Sarro, Cristina Segura-Garcia, Pasquale De Fazio

**Affiliations:** ^1^Psychiatry Unit, Department of Medical and Surgical Sciences, University “Magna Graecia” of Catanzaro, Catanzaro, Italy; ^2^Psychiatry Unit, Department of Health Sciences, University “Magna Graecia” of Catanzaro, Catanzaro, Italy; ^3^Department of Health Sciences, University “Magna Graecia” of Catanzaro, Catanzaro, Italy

**Keywords:** caregivers, cognitive dysfunction, COVID-19, dementia, pandemic, psychological symptoms, SARS-CoV-2, systematic review

## Abstract

**Background:** Coronavirus Disease 2019 (COVID-19) is a worldwide public health concern. It continues to spread rapidly throughout the world causing multiple physical and psychological consequences in the population. Especially, people affected by severe psychiatric or neurological diseases are highly susceptible to serious health complications not only due to the direct effect of the infection but also to the indirect effect of COVID-19 following social distancing during lockdowns and its general social consequences. Indeed, lockdown and difficulties in using the care services produced psychological consequences in caregivers such as depression, anxiety, and worsening of the quality of life which in turn affected the ability to manage patients. Our aim was to systematically review the psychological consequences of the COVID-19 lockdown in caregivers of patients with cognitive impairment and dementia and the impact on the health of their patients.

**Methods:** A systematic literature search was conducted by searching in MEDLINE/PubMed, Scopus, and Web of Science by two independent researchers following the Preferred Reporting Items for Systematic Reviews and Meta-Analyses (PRISMA) statement guidelines. Data extraction and quality assessment were also performed. Papers were screened for eligibility by abstract and then those which met inclusion criteria were included in this review.

**Results:** The initial search returned 410 records. After the abstract screening and the inclusion/exclusion criteria were applied, 315 were excluded because they were irrelevant, 30 because they were reviews, meta-analyses, letters to editors, editorials, guidelines, or case reports, and 10 because they were duplicates. Then, 38 out of 55 abstracts/full-text articles were excluded because they did not simultaneously assess mental health of patients and caregivers. In the end, 17 papers were deemed eligible and included in the present review.

**Conclusion:** Based on current literature, the COVID-19 pandemic and the ensuing lockdown caused severe psychological consequences for caregivers of patients with dementia, worsening their mental health, and increasing the psychological and physical burden, independently from the severity of the disease of their relatives, which resulted also independently globally worsened.

## Background

The severe acute respiratory syndrome coronavirus 2 (SARS-CoV-2) infection resulting in Coronavirus Disease 2019 (COVID-19) has caused the most severe health crisis since the 1918 influenza pandemic (Osuchowski et al., 2021). After the cluster of COVID-19 cases reported in China at the end of 2019, the virus has spread rapidly throughout the world, with over 200 million cases and 4 million deaths, with an unprecedented impact on healthcare systems, national economies, and society (Coronavirus Resource Center, 2021).

To contain the COVID-19 diffusion and avoid the overload of their medical systems, world government authorities have introduced restrictive measures based on lockdowns, travel limiting, social distancing, self-protection measures (e.g., wearing a face mask), and quarantine, which have caused negative psychological consequences in the population (Brooks et al., 2020; Clemente-Suárez et al., 2020).

The isolation that limits both individual movements and social contacts, the fear of contagion, the financial worries, and the loss of relatives or friends for COVID-19 has severely affected mental health wellbeing, increasing the risk of anxiety, depression, irritability, insomnia, and post-traumatic stress symptoms (Clemente-Suárez et al., 2020; Fofana et al., 2020).

On the other hand, it has been hypothesized that COVID-19 may induce a neuro-inflammatory state, triggering or accelerating neurodegeneration processes and related symptoms, such as neuropsychiatric manifestations (Iodice et al., 2021; Solomon, 2021), which in turn may further decline as a consequence of psychological symptoms due to stressor events (Song et al., 2020).

Taken together, this evidence suggests a greater vulnerability to COVID-19 sequelae for people already suffering from cognitive impairment (e.g., dementia, Parkinson's) who need a support network, such as caregivers and social and health services (Aamir et al., 2021). The changes in routine living conditions, such as the lockdown-related difficulties to access the care services, can deteriorate their mental and physical health. As a result, stress-related symptoms, depression, anxiety, and worsening quality of life may also occur in caregivers, impairing in turn, the ability to manage patients (Cagnin et al., 2020). Indeed, during the COVID-19 lockdown, caregivers of patients with severe neuropsychiatric diseases experienced an increased burden related to changes in neuropsychiatric symptoms of patients (Boutoleau-Bretonnière et al., 2020), and they also reported heightened feelings of responsibility as caretakers (Rising et al., 2021). In particular, it was demonstrated that female caregivers of patients suffering from neurocognitive disorders were affected by anxiety and depressive symptoms (Li et al., 2021). However, while dementia patients are often evaluated from both clinical and behavioral points of view, their caregivers are frequently neglected. Indeed, caregivers experience a double burden of both their loved one's care and their personal living with lockdown measures (Iodice et al., 2021; Numbers and Brodaty, 2021; Suárez-González et al., 2021). Finally, the impact of changes in the mental health status of caregivers on their patients adds to this already novel complicated situation; therefore, a systematic review focusing on the perspective of caregivers has long been needed.

Considering that this pandemic could be long-lasting and that the mental health of caregivers of patients with cognitive decline/dementia has been demonstrated to be at risk regardless of the COVID-19 emergency (De Fazio et al., 2015; Corrêa et al., 2019; Lloyd et al., 2019), there is a need to investigate the real effects of the current emergency on caregivers of patients with dementia to identify and address adequate interventions (Liu et al., 2021).

Our aim was to systematically review the psychological consequences of the COVID-19 lockdown in caregivers of patients with cognitive impairment and dementia and the impact on the health of their patients.

## Methods

### Literature Search

According to the Preferred Reporting Items for Systematic Reviews and Meta-Analyses (PRISMA) statement (Moher et al., 2009), a systematic search of the electronic databases MEDLINE/PubMed, Scopus, and Web of Science was performed from inception to August 1, 2021 by entering the following search string: (“COVID-19” OR “coronavirus disease” OR “coronavirus” OR “SARS-CoV-2” OR “novel coronavirus”) AND (“caregiver^*^” OR “relative” OR “Spouse^*^” OR “Adult Children” OR “Family” OR “Home Nursing” OR “career^*^” OR “caring” OR “caretaker” OR “home”) AND (“dement^*^” OR “Alzheimer's^*^” OR “mild cognitive impairment” OR “cognitive impairment” OR “cognitive dysfunction” OR “neurocognitive impairment” OR “MCI” OR “Mild dementia” OR “Parkinson's”).

Two blinded investigators (EC and RF) independently conducted a literature search, title/abstract screening, and full-text review. They also hand-screened the reference list of selected articles in order to search for additional literature. In case of disagreements, a final decision was achieved consulting a third investigator (RR).

### Study Selection

Original studies investigating psychological consequences of the COVID-19 lockdown in caregivers of patients with cognitive impairment and dementia and the impact on health of patients were deemed eligible for inclusion, according to the population, intervention, control, and outcomes (PICO) methodology. No time and language limits were used. Articles presenting only an opinion, reviews, letters to the editor, and commentaries were excluded. Studies evaluating only the point of view of patient or caregiver were not deemed eligible for the present review.

### Quality Assessment

The two reviewers (EC and RF) used the Grading of Recommendations, Assessment, Development, and Evaluation (GRADE) approach to rate the quality of the evidence (Guyatt et al., 2011). The quality of evidence was rated as “high,” “moderate,” “low,” or “very low” based on GRADE rating standards. A “high-quality” rating indicates that future research is very unlikely to change existing evidence and that the true effect is similar to the estimated effect; a “moderate-quality” and a “low-quality” rating indicates that future research may change/is likely to change the evaluation results, respectively; a “very low-quality” rating indicates that there is high uncertain about the existing evidence and that the true effect is likely to be substantially different from the estimated effect.

### Data Extraction

Two blind researchers (EC and RF) performed data extraction from included papers: first the name of the author, year of publication, study design, study sample, study measures, outcome, comments (limitations), timeline, and level of Grade. The reviewers independently extracted data from each relevant study and all reviewers checked the data for disagreement.

## Results

### Search Results

**Figure 1:**
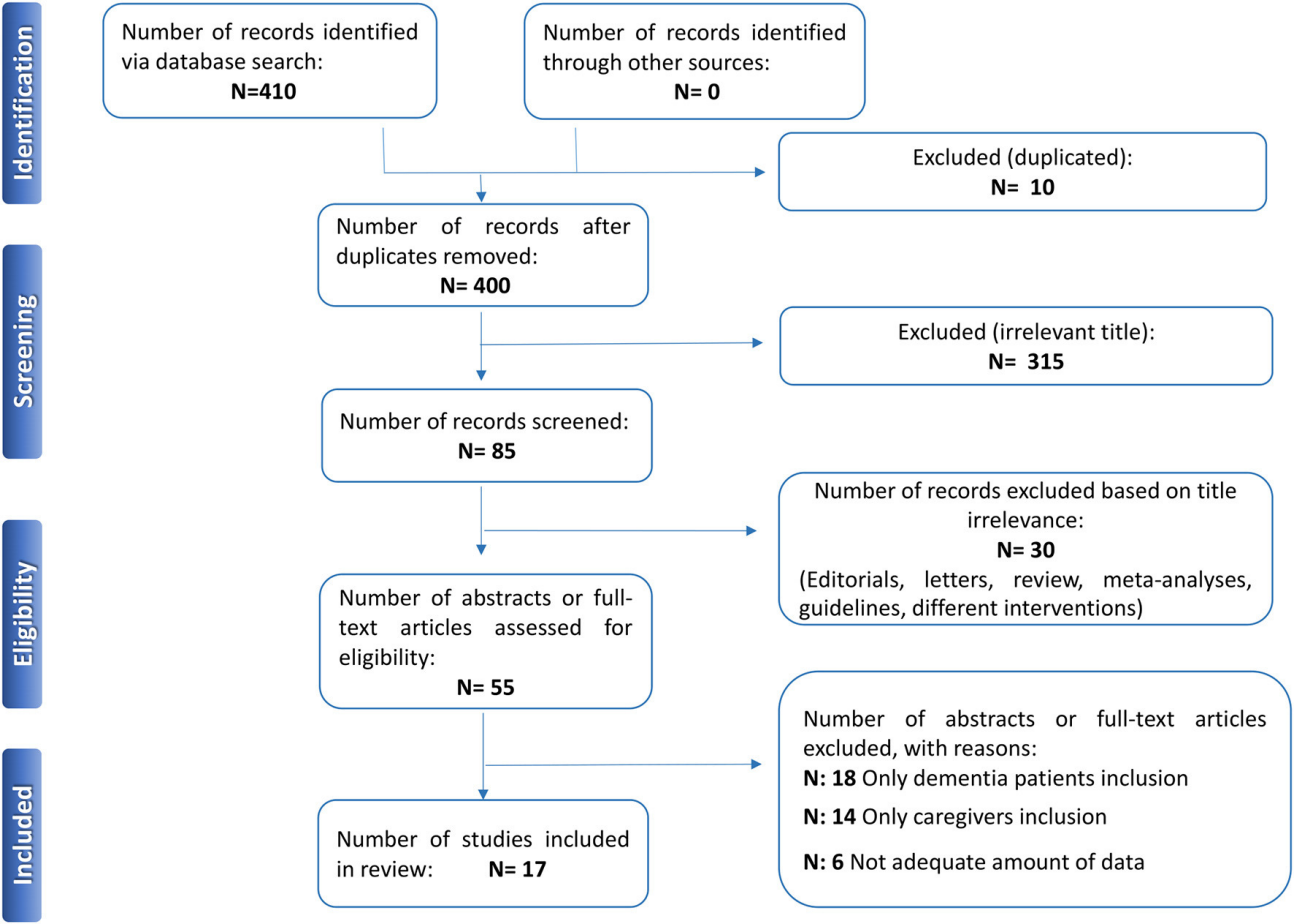
PRISMA flow-chart. PRISMA, preferred reporting items for systematic reviews and meta-analyses.

The initial search returned 410 records. Among these, 10 were identified as duplicates and were subsequently excluded. The title and abstract screening, based on the assessment of the inclusion/exclusion criteria, was performed for the remaining 400 papers; 315 were excluded because they were irrelevant and 30 because they were reviews, meta-analyses, letters to the editors, editorials, guidelines, or case reports. Then, 55 abstracts/full-text articles were assessed for eligibility: 38 were excluded because they did not simultaneously assess mental health of patients and caregivers. In the end, 17 papers were deemed eligible and included in the present review. The flow chart shows the search strategy (Figure 1).

### Content Results

The majority of the studies presented a cross-sectional observational qualitative study design. One was a multicentric study (Rainero et al., 2021), and only one study was a controlled non-randomized interventional prospective study (Lai et al., 2020). A great heterogeneity was reported among studies included and there was wide variability in the sample number from *N* = 5,321 caregivers and 4,913 patients (Rainero et al., 2021) to 11 carers and four patients (West et al., 2021). Online self-report questionnaires or interviews were conducted remotely by telephone or video call in almost all studies included and applied both to patients and family caregivers. The assessment of participants was performed using Montreal Cognitive Assessment (MoCA), the Revised Memory and Behavior Problem Checklist (RMBPC), and the Quality of Life in Alzheimer's Disease (QoL-AD) (Lai et al., 2020). Zung's depression (SDS) and anxiety (SAS) assessment scales and the Perceived Stress Scale (PSS) (Carpinelli Mazzi et al., 2020), the Short Warwick-Edinburgh Mental Well-Being Scale (SWEMWBS), the Generalized Anxiety Disorder 7 (GAD-7), the Personal Health Questionnaire 9 (PHQ-9) (Giebel et al., 2020b), Unified Parkinson's Disease Rating Scale Part II, Non-Motor Symptoms Scale (NMSS), Questionnaire for Impulsive-Compulsive Disorders in Parkinson's Disease–Rating Scale (QUIP-RS), and Parkinson's Disease Questionnaire-8 (PDQ-8). Hospital Anxiety and Depression Scale (HADS) scores were retrieved in patients and caregivers, who also underwent the Zarit Burden Interview (ZBI) (Oppo et al., 2020). HADS was used to assess anxiety and depression. The physical component summary (PCS) and mental component summary (MCS) scores of the short form (SF)-8 were used to evaluate health-related quality of life (QoL) (Suzuki et al., 2021), Neuropsychiatric Inventory Questionnaire (NPI-Q), ZBI, Beck Depression (BDI), and Anxiety (BAI) Inventories (Borelli et al., 2021). All the studies were conducted during the first lockdown, in different countries.

According to the GRADE approach, only one study presented high quality (Rainero et al., 2021) and three a moderate quality (Lai et al., 2020; Giebel et al., 2020b; Macchi et al., 2021), whereas most of the included research showed very low-to-low quality. Cross-sectional study design, not validated scales, self-report measures, and video or telephone interviews were the encountered limitations.

Four studies reported an increased level of anxiety, depression, and insomnia among caregivers, due to mandatory confinement and increased stress but also independently by the dementia stage, but globally those caring for severe cases had more stress compared to milder forms of the disease (Cohen et al., 2020; Rainero et al., 2021; Tsapanou et al., 2021).

Carpinelli Mazzi et al. found that education was a protective factor against anxiety and depression for caregivers, whereas women reported higher anxiety and depression levels (Carpinelli Mazzi et al., 2020). Moreover, Sukuzi et al. evaluated a negative impact of the COVID-19 pandemic on health-related QoL and its determinants in Parkinson's patients and their caregivers (Suzuki et al., 2021).

Overall, during COVID-19 confinement, caregivers reported a great increase in their psychological and physical burden, and patients with dementia experienced increased anxiety and an overall decline. The outbreak period led to a marked reduction of access to health services and social support services (Tam et al., 2021). The only positive feature was recorded thanks to the use of technology: telemedicine by videoconference was associated with improved resilience and wellbeing to both people with neurocognitive diseases and their caregivers at home compared to the telephone-only group (Lai et al., 2020) (Table 1).

**Table 1:** The main characteristics of included studies in the review.

**Author, year**	**Study design**	**Study sample**	**Measures**	**Outcome**	**Comments**	**Timeline**	**Grade**
Cohen et al. (2020)	Cross-sectional non-interventional study	80 family caregivers of AD or related dementia patients	Online questionnaire based on a visual analog scale to test burden	- Family was the primary provider of care in 65% - COVID-19 confinement increased caregiver's stress independently of the dementia stage, but those caring for severe cases had more stress compared to milder forms of the disease - 50% patients with dementia experienced increased anxiety - Most family members discontinued all sort of cognitive and physical therapies - Family members' main concerns: - Severe dementia case: fear of absence of the paid caregiver during the epidemic - Mild dementia cases: fear of spreading the disease while assisting patients	Limitations: - Small sample size - *p*-values not adjusted for multiple comparisons - Possible high rates of type I error - No validated instruments to measure burden of care or anxiety	Argentine—April 2020—first lockdown	^*^
Tsapanou et al. (2021)	Cross-sectional non-interventional study	204 caregivers of people with MCI or dementia	Self-reported questionnaire for caregivers regarding the status of patients and their own. Domains: changes in physical, psychological and routine activities with possible answers: “not at all,” “some” and “a lot”	- Significant overall decline of the people with MCI/dementia - Main decline in: communication, mood, movement, and compliance with the new measures - Caregivers reported a great increase in their psychological and physical burden	Limitations: - Self-reported measure, small sample size - Short period of time for the results reported - Results might be affected by the caregiver's increased workload •Strengths: - First study regarding elder Mediterranean population including specific questions about the patients with mci/dementia and their caregivers	Greece, Feb-June 2020—first lockdown	^**^
Lai et al. (2020)	Controlled not randomized interventional prospective study	60 dyads of elderly NCD patient-caregiver recruited through an activity center	Neurocognitive functioning, behavioral and psychological problems, and QoL were assessed in the care-recipient with NCD by MoCA, RMBPC, and QoL-AD	- Telemedicine by video conference was associated with improved resilience and wellbeing to both people with NCD and their caregivers at home compared to the telephone-only group	Limitations: - No head-to-head comparison between video conferences and phone calls with matching contact time - The switch from phone calls to video conference likely had affected the content, style, and manner of the delivery by the health care providers, and these should have been recorded and subjected to analysis to isolate potential mediator variables	Hong-Kong—March-May 2020—first lockdown	^***^
Carpinelli Mazzi et al. (2020)	Cross-sectional observational study	239 caregivers of patients with dementia	SDS, SAS, and PSS by telephone interview or online self-compilation	- Education was a protective factor against anxiety and depression for caregivers - Length of isolation was associated with the higher SAS and SDS scores. - Women reported higher SAS and SDS mean scores than men - A marked reduction of health services was observed in all patients	Limitations: - SAS not validated in Italian language - No validation of any of the measures for telephone interviews	Italy, first lockdown	^**^
Giebel et al. (2020a)	Cross-sectional observational qualitative study	Unpaid carers (*n* = 42) and PLWD (*n* = 8)	The semi-structured interviews were conducted using a topic guide, containing questions about the participant's service use before and after the COVID-19 outbreak and governmental restrictions	- A significant reduction in social support service usage since the outbreak emerged - Thematic analysis identified three overarching themes: loss of control, uncertainty, and adapting and having to adapt to the new normal - Carers and PLWD were greatly affected by the sudden removal of social support services, and concerned about when services would re-open - Carers worried about whether the person they cared for would still be able to re-join social support services	Limitations: - Sample size heterogeneity and number; not validated assessment	UK—April 2020—first lockdown	^*^
Giebel et al. (2020b)	Cross-sectional observational qualitative study	569 participants completed the survey (61 people with dementia, 285 unpaid carers, and 223 older adults)	SWEMWBS, GAD-7, PHQ-9	- Higher variations in social support service hours significantly predicted increased levels of anxiety in people with dementia and older adults, and lower levels of mental well-being in unpaid carers and older adults	Limitations: - Sample size heterogeneity and number - Patient enrollment procedure	UK—April-May 2020—first lockdown	^***^
Oppo et al. (2020)	Cross-sectional observational qualitative study	32 patients with PD/caregiver dyads	- Patients: UPDRS, NMSS QUIP-RS, and pDQ-8 - Carers: ZBI - Patients and carers: HADS and verbal rating scale (0–6) to measure changes in stress levels	- Patients experiencing increased stress level during lockdown had worse NMSS and HADS scores - Worse NMSS in patients associated to higher stress among carers - UPDRS-II score not associated with higher stress levels in patients/carers - Patients with increased stress had significant worse mood/cognition score of NMSS	Limitation: - Small sample size and the use of telephone interviews - Use of a non-validated outcome measure (verbal rating scale)	Italy—not provided 2020—first lockdown	^**^
Rainero et al. (2021)	Cross-sectional observational qualitative multicentric study	−5,321 caregivers of patients regularly followed - 4,913 patients with dementia (3372 AD; 360 DLB; 415 FTD; 766 VD)	- Semi-structured, self-made interview gathering demographic and clinical data from patient and caregiver - CDR	- According to family caregivers, social isolation, and physical restraint caused a worsening in cognitive function (55% of patients, mainly DLB and AD), an aggravation of several behavioral symptoms (52% patients), and a worsening in motor function (37% patients) and onset of new symptoms (26% patients) - Caregivers reported a high increase in anxiety, depression, distress, and burden	Limitation: - Data only regard patients with dementia living at home (data cannot be generalized to institutionalized patients) - Cross-sectional study - Not possible to administer face-to-face standardized neuropsychological - Tests	Italy—April 2020—first lockdown	^****^
West et al. (2021)	Cross-sectional observational qualitative study	15 participants: 11 family carers and four persons living with dementia	Semistructured qualitative interviews conducted remotely over telephone or via secure video technologies	- Eight key themes, with subthemes: fear and anxiety, food and eating (encompassing food shopping and eating patterns), isolation and identity, community and social relationships, adapting to COVID-19, social isolation and support structures, and medical interactions - Fear and anxiety formed an overarching theme that encompassed all others	Limitation: - Study focused on south Asian and afro-Caribbean groups and views may not be generalizable to other minority groups. •Strengths: - Study of BAME communities, including persons with dementia and their carers - Analysis performed by a different group - Analysis with an iterative constructivist approach and thematically codification	UK—May 2020—first lockdown	^*^
Tuijt et al. (2021)	Cross-sectional observational qualitative study	30 people with dementia living in their own homes and 31 family carers	Interviewed via video or telephone call	- Five main themes: awareness of restrictions, restructuring caring relationships to manage COVID-19 risk, protective factors, the psychological and cognitive impact of restrictions, and the importance of social engagement - People living with dementia often had a basic understanding of COVID-19 restrictions but could have difficulty translating this into personalized risk-appraisal of their own actions - Patients reported negative psychological and cognitive effects due to the imposed restrictions (e.g., increased apathy, irritability, or anxiety) fuelled by lack of social engagement	Limitation: - Difficulty communicating through telephone or video calls	UK—March-July-2020—first lockdown	^**^
Suzuki et al. (2021)	Cross-sectional observational qualitative study	100 patients with PD and their caregivers/spouses	HADS and SF-8	The study reveals the negative Impact of the COVID-19 pandemic on health-related QoL and its determinants in PD patients and their caregivers	Limitation: - Retrospective questionnaire-based - Changes in motor symptoms after the outbreak of COVID-19 were not assessed by means of clinical examination by neurologists	Japan, June and December 2020—first lockdown	^**^
Azevedo et al. (2021)	Cross-sectional observational qualitative study	321 dyadic interviews were conducted to patients and caregivers	Two semi-structured questionnaires via telephone to family caregivers of people diagnosed with dementia	- Significant decline in memory function among 53% of people with dementia - 31% of individuals with dementia felt sadder and 37% increased anxiety symptoms. Symptoms of anxiety were greater in individuals with mild to moderate dementia; symptoms of agitation were greater in individuals with severe dementia - Compulsive-obsessive behavior, hallucinations, increased forgetfulness, altered appetite, and increased difficulty in activities of daily living were reported more frequently among individuals with moderate to severe dementia - Caregivers reported feeling more tired and overwhelmed and these symptoms were also influenced by the severity of dementia	Limitation: - Interviews carried out by telephone - Countries experiencing different moments of the pandemic	Argentine, Brazil, Chile—May to July 2020—first lockdown	^**^
Tuijt et al. (2021)	Cross-sectional observational qualitative study	30 patients living with dementia and 31 carers	Semi-structured interviews with a background in psychology and dementia conducted remotely by telephone or video call	- The following three themes were identified: - Proactive care at the onset of COVID-19 restrictions - Avoidance of healthcare settings and services - Difficulties with encounters - People living with dementia and their carers felt check-up calls were reassuring but limited in scope and content. Some avoided healthcare services, wishing to minimize COVID-19 risk or reduce NHS burden, or encountering technological barriers - Difficulties in remote consultations included lack of prompts to remember problems, dealing with new emerging difficulties, rescheduling/missed calls, and inclusion of the voice of the person with dementia	Limitations: - Sample size - The median year of diagnosis (2019) was relatively recent	UK, May-August 2020—first lockdown	^*^
Macchi et al. (2021)	Multicenter, clinical trial of community-based	108 patients with PD, AD or related disorders and 90 caregivers	Semi-structured interviews, open-ended survey responses, medical record documentation, and participant-researcher communications	- Four main themes emerged: disruptions to delivery of healthcare and other supportive services; increased symptomatic and psychosocial needs; increased caregiver burden; and limitations of telecommunications when compared to in-person contact These themes interacted and intersected	Limitation: - Cohort lacks diversity regarding race, ethnicity, and was highly educateds	USA, March-August 2020—first lockdown	^***^
Hanna et al. (2021)	Cross-sectional observational qualitative study	4 PLWD and 16 unpaid carers	Semi-structured, follow-up telephone interviews	- Three primary themes emerged: impact on mental health during lockdown; changes to mental health following easing of public health; and the long-term effect of public health measures - The loss of social support services was key in impacting this cohort mentally and emotionally, displaying a need for better psychological support, for both carers and PLWD	Limitation: - Sample size and few people from BAME background - Interviews could not be conducted face-to-face	UK, June-July 2020—first lockdown	^*^
Borelli et al. (2021)	Cross-sectional observational qualitative study	58 patients and caregivers	A structured telephone interview with NPI-Q, ZBI, BDI and BAI	- Frequent patients' neuropsychiatric worsening and caregiver burden - Worsening of cognition was associated with increased caregivers' psychological distress	Limitations: - Cross sectional design of the study - Lack of previous caregiver scores in the scales may overestimate the pandemic's impact	Brazil, May-July 2020—first lockdown	^**^
Tam et al. (2021)	Cross-sectional observational qualitative study	395 care partners and 22 individuals with lived experiences of dementia	Survey	- Care partners reported a number of serious concerns, including the inability to visit the person that they care for in long-term or palliative care - The pandemic increased their levels of stress overall and felt lonelier and more isolated than they did before the pandemic - The use of technology was reported as a way to connect socially with their loved ones, with the majority of participants connecting with others at least twice per week	Limitations: - Cross sectional study design	Canada, June-August 2020—first lockdown	^**^

*AD, Alzheimer's disease; BAME, Black, asian and minority ethnic; BPSD, Behavioral and psychological symptoms of dementia; CDR, Clinical dementia rating; DLB, Dementia with lewy bodies; FTD, Frontotemporal dementia; MCI, Mild cognitive impairment; MoCA, Montreal cognitive assessment; NCD, Neurocognitive decline; NMSS, Non-motor symptoms scale; PD, Parkinson's disease; PDQ-8, Parkinson's disease questionnaire-8, PLWD, Person living with dementia; PSS, Perceived stress scale; QoL, Quality of life; qol-ad, Quality of life in alzheimer's disease; QUIPRS, Questionnaire for impulsive-compulsive disorders in parkinson's disease–rating scale; RMBPC, Revised memory and behavior problem checklist; SAS, Zung's self-rating anxiety scale; SDS, Zung's self-rating depression scale; SWEMWBS, Short warwick-edinburgh mental well-being scale; UPDRS, Unified parkinson's disease rating scale; VD, Vascular dementia; ZBI, Zarit burden interview*.

*Grade score:*

* *Very low quality*,

** *Low quality*,

*** *Moderate quality*,

**** *High quality*.

## Discussion

This systematic review identified 17 studies that provided dyadic information on psychological symptoms of family caregivers of patients with cognitive impairment or dementia due to different causes and in various stages of dementia living in the community and the effects in these subjects and on the health of patients during the first COVID-19 lockdown.

The United Kingdom (UK) and Italy contributed with the greatest number of studies and patients, respectively. Therefore, despite remaining studies also representing Asian and American populations, and a small UK study aimed to specifically investigate the impact of the COVID-19 pandemic on black, Asian, and minority ethnic populations (West et al., 2021), results are hardly generalizable.

Indeed, data on all minority groups are not available, and socio-economic, cultural, educational, and setting factors should be considered also in studies with a larger sample size. As an example, in the largest study included in this review (Rainero et al., 2021), caregivers of patients with dementia reported a significant increase in anxiety, depression, irritability, distress, and an overall clinical worsening of their loved ones, although these results cannot be referred to caregivers of institutionalized patients.

Moreover, as in most studies included, no validated scales were used to measure psychological symptoms. In other studies, although they were used, the scales administered had not been previously validated either in the translated version or to be used as telephone interviews (Carpinelli Mazzi et al., 2020). Suzuki et al. used stepwise linear regression analysis to identify key predictors of health-related QoL after having administered validated scales for anxiety, depression, and QoL (Suzuki et al., 2021). This study, limited by the retrospective nature, showed a negative impact of depression, stress, and worsening patient mood on mental aspects of the health-related QoL in caregivers/spouses of patients with Parkinson's disease (Suzuki et al., 2021). Validated outcome measures were also used to describe an association between the worsening of cognition in patients with dementia and the increasing burden and distress in their caregivers (Borelli et al., 2021), as well as anxiety and worsened QoL associated with the inability to access social support services (Giebel et al., 2020b).

On the other hand, additional information emerged from semi-structured qualitative interviews and open-ended surveys, showing the perspective of caregivers, their feelings, and main concerns, which might suggest potential areas of intervention to address unmet social and healthcare needs.

During this social distancing period, approximately half of the family caregivers felt more tired, overwhelmed, and nervous, and more than a third felt sadder and more irritable (Azevedo et al., 2021). Furthermore, the dementia severity affected the burden experience that was perceived as greater in carers of people with severe dementia (Cohen et al., 2020; Azevedo et al., 2021). In these patients, agitation, cognitive, and behavioral symptoms were greater or more common compared with individuals with mild-to-moderate dementia (Azevedo et al., 2021). Moreover, caregivers of patients with mild cognitive impairment or dementia referred to an increase of their own burden together with a significant decline of overall patients, although a self-reported questionnaire was used and statistical association measures were not performed between these two variables (Tsapanou et al., 2021).

Feelings of stress attempting to take care of their loved ones and the negative impact on the mental and emotional wellbeing of the loss of social support services were also reported in small sample size studies, suggesting the need for better psychological support (Hanna et al., 2021). Care partners felt less able to manage their own wellbeing and reported burnout and worries about both their increasing workload and the infection risk of patients with dementia (Tam et al., 2021). They were also concerned about the faster deterioration of their relatives, which is partly real (social isolation and physical restraint worsen cognitive and motor function), partly biased by the overload, and a heightened awareness assessing health needs (Canevelli et al., 2020; Tuijt et al., 2021).

Additionally, the negative impact of restrictive measures on the autonomy of patients with dementia and on all aspects of caregivers' lives increased pressure on caring relationships (Tuijt et al., 2021).

One of the few moderate quality studies according to the GRADE approach provided a person-centered description of the impact of the COVID-19 pandemic, whose results can be considered an exhaustive overview of the main themes that also emerged from other semi-structured interviews and open-ended surveys (Macchi et al., 2021). Besides the increased caregiver strain, disruption to the delivery of healthcare and supportive services was deeply perceived. This latter included cancellations of routine appointments and elective procedures, loss of ambulatory services and home health aides, and avoidance of assisted living facilities to avoid contagion. Moreover, increased symptomatic and psychosocial needs, as well as the indispensable role of in-person contact and support emerged (Macchi et al., 2021). Routinely used social support services and facilities are known to improve wellbeing and quality of life of dementia patients (Greenberg et al., 2020). The limited access/suspension of essential services due to COVID-19 contributed to worsening psychological symptoms and quality of life, especially for patients with more routine social activity outside the home before the pandemic (Tuijt et al., 2021).

Along with dementia severity, type of symptoms (especially neuropsychiatric and autonomic, such as sleep disturbances) and prolonged time of isolation may have impacted stress level, anxiety, and depression of caregivers (Carpinelli Mazzi et al., 2020; Oppo et al., 2020). These were higher in female caregivers compared with males (Carpinelli Mazzi et al., 2020). Women represented the majority of caregivers (up to 80% of the carers population) (Azevedo et al., 2021), as confirmed in epidemiological data (Alzheimer's Society, 2020); socio-economic, health, and psychosocial factors contribute to gender differences in psychological wellbeing (Kiely et al., 2019). As an example, the educational level of carers seemed to represent a protective factor (Carpinelli Mazzi et al., 2020).

In addition to potential protective factors, literature evidenced many strategies to relieve stress of caregivers, such as avoiding isolation, sharing the burden of care with other family members, and support networks (Hughes et al., 2014). Indeed, in ethnic minorities in which more established familial care structures existed, changes to living arrangements due to COVID-19 did not increase the pressure of carers, although a likely greater difficulty in accessing support services emerged (Tuijt et al., 2021).

However, these findings should be interpreted bearing in mind the very low to low quality of evidence of the majority of the studies included and a large number of limitations.

Although the studies' selection ensured a specific dyadic evaluation of patients and caregivers, which allowed, on the one hand, a deeper comprehension of both settings and mutuality of the care relationship and on the other a reduction of heterogeneity of studies, this latter still remains, as well as a wide variability in the sample number. Contrariwise, the lack of variability of the sample in terms of represented populations negatively affects generalization of the findings.

Besides these issues and no validated outcome measures, qualitative research and self-reported data through video or telephone interviews affected the quality of the results. Indeed, the interviews may have been influenced by the emotional state of the caregivers on the day of the survey, and by the role and position of the researcher who conducted the interviews, communication, and connection difficulties (Bauhoff, 2011). Furthermore, video or telephone interviews are clearly compromised by a selection bias, under-representing people with difficulties accessing technologies. Finally, almost all the papers included in this review had a cross-sectional design, which does not allow for comparison with baseline data (i.e., before the COVID-19 period). Therefore, it is impossible to quantify the overall increase in psychological and physical burden of caregivers emerging from studies. Independently from the pandemic, caring for patients with dementia is a complex task associated with significant burden and distress, which increase as the disease progresses and cognitive, behavioral, and motor symptoms worsen (Chiao et al., 2015; Sutcliffe et al., 2017; Black et al., 2018). Thus, it would be useful to compare results at different time points and observe the effects of isolation and social distancing over time. The only prospective study included showed the positive impact of supplementary telehealth delivered via video-conferencing apps compared with phone calls alone (Lai et al., 2020). Remote support to examine patients has been validated in several studies (Roy et al., 2021) although a general reluctance among practitioners to implement alternatives to face-to-face consultations was reported (Brant et al., 2016), and patients and caregivers did not perceive it as a substitute for in-person services (Macchi et al., 2021; Tam et al., 2021). Video conferences may overcome these limitations capturing some typical aspects of the face-to-face interaction, suggesting that improving remote support and using communication technologies could represent a new way through which health and social support services can aid. To date, telemedicine is not widely applicable yet: a large number of people do not have access to this resource or the ability to use them.

## Conclusions

Despite the very low-to-low quality of evidence and several methodological limitations of some studies included in this review (not allowing a sound estimation of our aim), results suggest a great increase in psychological and physical burden of caregivers during COVID-19 confinement and an overall worsening of clinical conditions of patients. This analysis indicates that the perceived burden in caregivers increased independently from the severity of their relatives' disease, albeit it was apparently higher for those caring for severe cases compared with milder. Furthermore, gender represents a risk factor and supporting strategies, such as telemedicine, likely improve outcomes of caregivers and patients.

Restrictive measures led to a marked reduction of health and social support services, which added new burdens and/or intensified pre-existing ones. Better psychological support and new healthcare strategies, such as the implementation of telemedicine and digital communications (addressing technological barriers), are needed to provide assistance, ensure a supportive network, and relieve the stress of caregivers.

Further high-quality studies are necessary, and prospective data will help to monitor any change in social and health needs in patients and carers. Moreover, data on heterogeneous samples for ethnicity, cultural and socio-economic conditions, and kind of care relationships should be encouraged to meet specific needs and direct healthcare resources throughout the pandemic.

## Data Availability Statement

The original contributions presented in the study are included in the article/supplementary material, further inquiries can be directed to the corresponding author.

## Author Contributions

PDF ideated the manuscript. EAC and RdF conducted the literature search and data analysis. EAC, RdF, RR, MR, LD, ER, GDS, CS-G, and PDF contributed to writing the original draft and to its reviewing and editing. All authors contributed to the article and approved the submitted version.

## Conflict of Interest

The authors declare that the research was conducted in the absence of any commercial or financial relationships that could be construed as a potential conflict of interest.

## Publisher's Note

All claims expressed in this article are solely those of the authors and do not necessarily represent those of their affiliated organizations, or those of the publisher, the editors and the reviewers. Any product that may be evaluated in this article, or claim that may be made by its manufacturer, is not guaranteed or endorsed by the publisher.

## Abbreviations

BAI: beck anxiety inventory
BDI: beck depression inventory
COVID-19: coronavirus disease 2019
GAD-7: generalized anxiety disorder 7
HADS: hospital anxiety and depression scale
MCS: mental component summary
MoCA: montreal cognitive assessment
NMSS: non-motor symptoms scale
NPI-Q: neuropsychiatric inventory questionnaire
PCS: physical component summary
PDQ-8: parkinson's disease questionnaire-8
PHQ-9: personal health questionnaire 9
PSS: perceived stress scale
QoL: quality of life
QoL-AD: quality of life in alzheimer's disease
QUIP-RS: questionnaire for impulsive-compulsive disorders in parkinson's disease–rating scale
RMBPC: revised memory and behavior problem checklist
SARS-CoV-2: severe acute respiratory syndrome coronavirus 2
SAS: zung's self-rating anxiety scale
SDS: zung's self-rating depression scale
SF-8: short form health survey
SWEMWBS: short warwick-edinburgh mental well-being scale
ZBI: zarit burden interview.
